# The Impact of the COVID-19 Pandemic on Male Intimate Partner Violence Victims

**DOI:** 10.3390/bs16050707

**Published:** 2026-05-05

**Authors:** Denise A. Hines, Elizabeth A. Bates, Julia Taylor

**Affiliations:** 1 Department of Social Work, College of Public Health, George Mason University, Fairfax, VA 22030, USA; 2 Institute of Health, University of Cumbria, Carlisle CA1 2HH, UK; elizabeth.bates@cumbria.ac.uk; 3 Joshua Tree Psychotherapy, Sandy, UT 84094, USA; julia.jane.taylor@gmail.com

**Keywords:** male victims, domestic violence, pandemic, help-seeking, barriers, intimate partner violence

## Abstract

The COVID-19 pandemic contributed to more severe and frequent intimate partner violence (IPV) among victims, and less availability of services; however, this research has largely been conducted on only female victims. We investigated the COVID-19 pandemic’s contribution to more severe and frequent IPV among male victims, barriers to getting help, and factors contributing to both increased severity/frequency and barriers. Participants included 318 male IPV victims from English-speaking Western countries who reported being the victim of IPV during the pandemic. They completed a Qualtrics questionnaire asking about their IPV experiences, mental health, COVID-19-related experiences in general and IPV experiences in specific. Overall, 47.8% of the participants experienced an increase in frequency and/or severity of IPV victimization, with help-seeking barriers, job loss, being confined to the house with their aggressor, and prior trauma independently predicting increases. Also, 75.5% reported one or more barriers to accessing help; such barriers were independently predicted by increased severity/frequency of IPV, financial strain, relationship length, being married, using marijuana, severe depression, prior trauma, IPV stigma, and coercive control victimization. Results are discussed in terms of their consistency with the literature on female victims, and the need for gender inclusive research, service provisions, and service recommendations in light of crises such as the COVID-19 pandemic.

## 1. Introduction

Intimate partner violence (IPV) is a well-known public health issue that includes physical violence, sexual violence, stalking, and psychological aggression by a current or former intimate partner (Breiding et al., 2015). Specifically, IPV can include acts such as slapping, punching, choking, and use of a knife or gun (physical violence); rape, sexual coercion, and unwanted sexual contact (sexual violence); and insulting or humiliating an intimate partner and controlling or monitoring an intimate partner (psychological aggression; Smith et al., 2018). Most research has focused on women as victims of IPV, even though it is known that men are also victims (Hines & Douglas, 2019; Smith et al., 2018). In fact, according to the National Intimate Partner and Sexual Violence Survey (NISVS) by the U.S. Centers for Disease Control, a substantial portion of IPV victims are men. Lifetime estimates from the NISVS indicate that approximately 46.1% of all individuals who experienced IPV—including contact sexual violence, physical violence, and/or stalking by an intimate partner—were men. Similarly, approximately 46.8% of all IPV victims in the previous year were men (Smith et al., 2018). Like IPV towards women, IPV towards men can have severe consequences, such as injuries (Khurana et al., 2021), cardiovascular health issues (Hines & Douglas, 2015), post-traumatic stress disorder symptoms (Hines & Douglas, 2011), and depression (Hines & Douglas, 2015). In addition, male victims of IPV face substantial barriers to accessing support, some of which stem from the persistent framing of women as the sole or primary victims of IPV. These barriers include stigma, fear of not being believed, limited availability of appropriate support services, provider biases that men cannot be victims, social isolation and lack of support, and concerns about retaliation (see Machado et al., 2017 for a review). Some of these barriers also overlap with barriers that female victims experience (e.g., Robinson et al., 2021). Despite this growing body of evidence, men’s IPV experiences remain understudied, particularly during periods of widespread societal disruption. Although gender-specific mechanisms may shape how IPV is experienced and navigated, the present study focuses on documenting men’s lived experiences, consequences, and barriers to support during the COVID-19 pandemic rather than testing gendered pathways.

Historically, during and after disease outbreaks, violence—including that between intimate partners—increases (Peterman et al., 2020). On 11 March 2020, the World Health Organization (WHO) declared the novel coronavirus (COVID-19) outbreak a global pandemic; an end to the pandemic was declared in May of 2023. In an attempt to slow the spread of the virus, governments around the world implemented safety protocols, such as the use of face coverings, social distancing, and home lockdown (Usta et al., 2021), particularly in the early stages of the pandemic before vaccines became more widely available. During this time of lockdowns and social distancing, there was a rise in IPV calls to hotlines and law enforcement services in countries around the world (Nesset et al., 2021; Peterman et al., 2020; Richards et al., 2021). Furthermore, due to social isolation practices, access to support services were diminished, contributing to the continuation and worsening of IPV for female victims (Fornari et al., 2021). There has been a proliferation of research on the impact of the COVID-19 pandemic and associated lockdowns on IPV victims’ experiences of abuse and access to resources, and this literature has almost exclusively focused on female IPV victims. The purpose of this paper is to explore the impact of the COVID-19 pandemic on male IPV victims.

### 1.1. Increases in IPV Associated with COVID-19

The circumstances during the height of the COVID-19 pandemic led to several conditions that research shows contribute to increased rates of IPV, such as social and physical isolation, economic stress, and psychological stress (Ceroni et al., 2023). More specifically, stay-at-home directives and social distancing recommendations resulted in forced cohabitation with abusive intimate partners, providing a foundation for increased IPV (Ceroni et al., 2023; Gosangi et al., 2021). These directives even mimicked common forms of IPV, such as controlling behaviors, in that they forced isolation from friends and family members, prevented the victim from working or attending to responsibilities outside of the home, and allowed perpetrators to more closely monitor the movements and activities of victims (Piquero et al., 2020). Thus, many victims of IPV were positioned to experience heightened violence, and in fact, lived through increased incidents of IPV (Campbell, 2020; McLay, 2022; Piquero et al., 2021). For some victims, the lockdowns associated with the pandemic interrupted plans to leave their abusive relationships because access to support became limited (Lyons & Brewer, 2022). In other words, the lockdowns associated with the COVID-19 pandemic resulted in diminished safety and greater risk for continued—and even increased—IPV by aggressive partners.

Further, implemented regulations during the pandemic resulted in closures of nonessential businesses and public services, contributing to loss of employment and economic instability (Ceroni et al., 2023). This, in turn, contributed to many households existing in poverty (Lyons & Brewer, 2022) and financial strain, low income, unemployment, career stress, and poverty are all associated with IPV (Matjasko et al., 2013). IPV has been linked to financial stressors, in that economic hardship decreases coping resources for couples while simultaneously creating an environment where IPV is more likely to occur (Lucero et al., 2016). In addition, financial strain can be used as a means of controlling victims by reducing the likelihood of them leaving the violent relationship (Lyons & Brewer, 2022). Along those same lines, financial stressors are associated with increased odds of making threats, minor physical IPV, severe physical IPV, and injury (Schwab-Reese et al., 2016). Specific to the COVID-19 pandemic, Schokkenbroek et al. (2021) found that individuals experienced heightened verbal IPV when their aggressive partner was unemployed, either long-term or temporarily, because of the pandemic.

After the onset of the COVID-19 pandemic, adults reported that the pandemic had increased mental health concerns, such as the onset of fear and anxiety related to exposure to the virus (Kirzinger et al., 2020). This provided aggressive partners the ability to use COVID-19 as a means of psychological control over victims (Ragavan et al., 2022; Sower & Alexander, 2021). Coercive control tactics included perpetrators threatening to expose their partner or loved ones to the virus, perpetrators faking COVID-19 symptoms, and perpetrators coughing in victims’ faces (Sower & Alexander, 2021). Thus, the pandemic created an environment wherein perpetrators of IPV found access to additional methods of IPV against their partners, resulting in increased problems related to the mental health of victims.

The current paper expands on the current research to explore whether IPV increased in frequency and/or severity among men who report IPV victimization, while also exploring potential correlates of increases, such as being subjected to stay-at-home orders, being confined to their homes with their aggressive partners, inability to access supports or support services, job loss, financial strain, career strain, and/or mental and behavioral health issues, all of which have been shown to increase IPV among female victims during the COVID-19 pandemic. In addition, we explored whether other aspects of the COVID-19 pandemic, such as having a job that exposes one to the virus, other background and demographic variables, and IPV variables were associated with increased frequency and/or severity of IPV among male victims.

### 1.2. Barriers to Accessing Resources During COVID-19

In addition to the myriad ways in which the COVID-19 pandemic directly contributed to both new forms of IPV as well as worsening IPV, it is important to note that traditional help-seeking resources were also limited during this time (Lyons & Brewer, 2022). Stay-at-home orders provided perpetrators the opportunity to increase surveillance and restrictions on their partners by monitoring their social media, internet, and cell phone usage, thus preventing them from seeking help through electronic means (Campbell, 2020). Similarly, due to government-issued lockdowns, the ability of victims to find a reprieve in locations outside of their home, such as libraries, schools, and churches, became limited (Campbell, 2020). Prior to the pandemic, victims had access to many services, such as using shelter and counseling services. During the COVID-19 pandemic, these support services were disrupted, such that practical support for victims was reduced, preventing victims from escaping to shelter services or even to friends and family (Lyons & Brewer, 2022). Additionally, supports not directly tied to IPV, such as medical and law enforcement services, were also disrupted by the effects of the pandemic, potentially limiting the contact that victims would otherwise have had with these groups (Lyons & Brewer, 2022).

Furthermore, informal supports, such as IPV victims’ friends and family members, also experienced reduced capacity to provide help during the pandemic. Just as victims adjusted to stay-at-home orders, their support systems did as well, adjusting to unexpected life barriers such as working from home, providing additional childcare, and helping with home-schooling, and as the rest of their lives became more demanding, some providers of informal support noted that their capacity to help was stretched and diminished so that they were unable to provide needed aid to victims (Gregory & Williamson, 2022). Additionally, fears of spreading or contracting the novel coronavirus also served to prevent both victims and their friends and family from connecting with one another in person, which may have been needed (Gregory & Williamson, 2022).

Nonetheless, the research on barriers and predictors of barriers among IPV victims during COVID-19 is still limited, with little to no research on male IPV victims. Thus, we will explore what barriers to seeking help, if any, that male IPV victims experienced during the COVID-19 pandemic. In addition, we will explore potential correlates of those barriers, such as COVID-19 job loss, worsening IPV experiences, and mental and behavioral health issues, as well as whether certain demographic categories may have experienced more barriers than others.

### 1.3. Summary, Hypotheses and Research Questions

Existing evidence suggests that the COVID-19 pandemic forced IPV victims to face unprecedented challenges. However, most of the evidence addresses only the experiences of female victims during the pandemic, and not those of male victims. It is imperative to understand the experiences of male victims during the COVID-19 pandemic to inform the future of prevention and intervention services for all victims of IPV. Thus, this study explored the extent to which male IPV victims experienced increasing frequency and/or severity of IPV because of the COVID-19 pandemic, and the extent to which they experienced various COVID-19 barriers to seeking help. We also explored various potential correlates of increasing frequency/severity of IPV victimization and barriers to accessing help, including COVID-19-related struggles, demographics, IPV experiences, and mental and behavioral health issues. Although most of our analyses were exploratory, we also hypothesized that the following variables would be associated with increasing frequency/severity of IPV victimization, based on the literature reviewed above on female victims: being subject to stay-at-home orders, being confined with one’s partner to home, barriers to getting access to support, job loss, financial strain, career strain, and mental health concerns.

## 2. Materials and Methods

### 2.1. Participants

The data for this study (*n* = 318) is a subsample of a larger study on male-identifying victims of IPV (*N* = 594). The larger study goal was to investigate men’s experiences with IPV, help-seeking, and mental health across various countries and contexts. Only participants who indicated that their IPV victimization happened sometime between March 2020 and November 2021 were included in the subsample for the current paper.

To be eligible for the larger sample, men had to be from an English-speaking Western country (United States, Canada, United Kingdom, Ireland, Australia, and New Zealand) and between the ages of 18 and 59. Additionally, participants had to answer affirmatively to a screener question that “at least one of their romantic partners acted aggressively towards them, tried to control them, or forced or coerced them into something they did not want to do at some point in their life.” A total of 594 participants qualified for the larger sample; please see Hines et al. (2025) for more information on sampling.

For the current analyses, we focused on participants (*n* = 318) who completed the COVID-19 questions, which were only presented to men who reported IPV victimization between March 2020 through whenever they completed the survey in 2021. We programmed the Qualtrics survey to only ask these questions to men who reported being in their relationship within the timeframe specified, based on demographic questions asking whether they were still in the relationship and, if not, how long ago it had ended in months. Thus, if they reported that they were currently in their aggressive relationship and/or if the relationship had ended after March 2020, they were included in the current sample. Table 1 presents the demographics of the male participants and their reported demographics of their partners. In addition to what is presented in Table 1, 79.9% (*n* = 254) men were from the United States, 6.9% (*n* = 22) were from England or Scotland, 0.9% (*n* = 3) were from Ireland, 4.4% (*n* = 14) were from Canada, 2.8% (*n* = 9) were from New Zealand, and 5.0% (*n* = 16) were from Australia.

### 2.2. Procedures

Data were gathered using an anonymous online survey in Qualtrics, which was distributed from February to November 2021. Participants were recruited via advertisements on social media sites and through ads sent to agencies and other professionals who specialize in issues either directly or indirectly related to male victims of IPV (e.g., domestic violence, mental health, health, divorce, and parenting). The ad stated that we were “conducting a study on men who experienced aggression from their romantic partners.” Men were offered the opportunity to be paid $10 in an Amazon gift card for their participation; to be paid, they were redirected to a different survey at the end of the study, where they could enter their email to be sent an electronic gift card. After providing consent, participants were presented with questions to assess the previously mentioned screening criteria. Men who did not meet the eligibility requirements were thanked for their time and were redirected to the end of the survey.

The methods for this study were approved by the boards of ethics at all participating institutions. All men in the sample participated anonymously, and steps were taken to ensure their safety. At the end of the survey, participants were given information about agencies in their country that could provide services if they experienced distress. Additionally, the men were provided with instructions on how to erase their internet history.

### 2.3. Measures

#### 2.3.1. Demographics

To complete the survey, participants were told to focus on their aggressive romantic partner; if they had more than one in their lifetimes, they were asked to focus on their most recent aggressive romantic partner. The sample for the current analysis includes only men who were reporting on a current (i.e., ongoing) or recent (i.e., the relationship ended after March 2020) aggressive romantic partner. Participants were asked basic demographic information about themselves and their aggressive partners, including age, race/ethnicity, education, employment status, sexual orientation, and gender identity. The men were also asked what their current relationship was with their aggressive partner, how long they had been in that relationship, and whether they had any children with their aggressive partner.

#### 2.3.2. Intimate Partner Violence

We used the Revised Conflict Tactics Scales (CTS2; Straus et al., 1996) to measure the extent to which the men ever sustained severe psychological, physical aggression, and injuries in their relationships with the aggressive partner they reported on in this study. The items used for this study included four items assessing severe psychological aggression (e.g., threatening to hit or throw something at a partner, calling a partner fat or ugly), 12 items assessing physical aggression (e.g., slapping, beating up), and six items assessing injuries (e.g., having a small cut or bruise, broken bone, passing out). We supplemented the CTS2 with ten items from the Psychological Maltreatment of Women Inventory (PMWI; Tolman, 1995) that focused on controlling behaviors and could be applied to men as victims (e.g., monitoring one’s time and whereabouts, not allowing one to see family and friends). A factor analysis (Hines & Douglas, 2010) showed that these items represented a unique factor that is distinct from the severe psychological aggression items of the CTS2. See Hines and Douglas (2010) for the specific items used. We also utilized the Threatened Legal/Administrative Aggression scale developed and validated on previous population-based and male victims samples by Hines et al. (2015). This scale contained six items, such as threatening to make false accusations to authorities that the partner physically or sexually abused the other.

Participants responded to all IPV items by indicating whether that act was used by them and/or their aggressive partner at any time in their relationship (i.e., yes/no response). For the current analyses, each scale (i.e., victimization of each type of PV) was scored in two ways: (1) Whether any of the types of aggression ever happened (dichotomous yes/no variable), and (2) The number of different acts of each type of aggression that ever happened (e.g., there were a total of 12 items of physical aggression, so participants could be victimized by up to 12 types of physical aggression). This method of scoring is called a variety score and is recommended by Moffitt et al. (1997), who showed that variety scores provide a reliable and valid assessment of the severity and frequency of the various forms of IPV, without violating statistical assumptions.

The CTS2, PMWI items, and Legal/Administrative Abuse scales have good validity and reliability (Hines & Douglas, 2010; Hines et al., 2015; Straus et al., 1996). Reliability statistics for the current sample were generally between 0.70 and 0.89, with some exceptions. Lower alpha reliability occurred for scales that had little variability in item response (i.e., almost everyone endorsed or did not endorse all items). Victimization from severe psychological aggression had the lowest alpha reliability at 0.61 and had high endorsement of the items. Highest alpha reliabilities were for the perpetration of physical IPV (0.89) and controlling behaviors (0.88). As per Novick and Lewis (1967), Cronbach’s alpha underestimates and does not accurately reflect reliability when there is limited variability on items and when items are measured dichotomously, as was the case in the current analyses.

#### 2.3.3. COVID-19 Questions

All men who reported IPV victimization in March 2020 through 2021 were asked specific questions pertaining to the coronavirus pandemic, based on the extant literature at the time of study development on IPV and the COVID-19 pandemic. Questions focused specifically on issues that could impact the frequency and severity of IPV and/or could impede a victim from seeking help. Participants were asked whether they were subject to stay-at-home orders/restrictions due to COVID-19 while they were with their aggressive partner. They were asked whether they were confined to their house with their aggressive partner during the pandemic, and if they were, they were asked to specify the length of time they were confined together. Additionally, the men were asked questions pertaining to both themselves and their partners: whether they and/or their aggressive partner experienced a job loss during the pandemic; whether they and/or their aggressive partner experienced a change in work schedule during the pandemic; whether they and/or their aggressive partner were designated as essential workers during the pandemic; whether they and/or their aggressive partner’s occupation put them at risk for infection of the coronavirus; and whether they and/or their aggressive partner fell ill with the coronavirus during the pandemic, and if so, whether they and/or their aggressive partner needed to be hospitalized.

In addition, the participants were asked two questions related to their finances: whether they felt that the coronavirus pandemic led to financial strain on themselves and/or their aggressive partner and whether the pandemic led to additional, unforeseen expenses. Participants were also asked whether they felt that their partner’s aggressive behavior became more severe and/or frequent during any stay-at-home orders/restrictions. The men were also asked questions about their children: whether they had school-aged children living with them; if so, did they move to fully remote learning due to the pandemic at any point during the pandemic; and, if the children living with them participated in childcare outside the home, if the pandemic prevented them from using that resource at any time during the pandemic. Finally, participants were asked if they felt that the pandemic prevented them from getting assistance to help stop their partner’s aggression. See Table 2 for the response options for this question.

#### 2.3.4. Mental and Behavioral Health Measures

Participants completed a variety of measures to assess their mental and behavioral health. Depression symptoms were measured with the Patient Health Questionnaire (PHQ9; Kroenke et al., 2001), which contains nine questions related to DSM-5 criteria for major depression over the previous two weeks. Response options range from 0 (not at all) to 3 (nearly every day). The PHQ9 has excellent reliability and validity. Scores range from 0 to 27, and a cut-point of 20 represents severe depression. Cronbach’s alpha for the current sample was 0.91. Substance Use was measured using an update of the scale developed for the National Women’s Study (Kilpatrick et al., 1997), one of the purposes of which was to assess the association between alcohol/drug abuse and IPV victimization among women. Participants answered questions about whether they ever used alcohol, marijuana, and illicit drugs, and whether they ever misused prescription medications. If they ever used alcohol, they were asked how frequently (0 = never, 7 = daily). This scale has shown excellent construct validity (Kilpatrick et al., 1997). Suicide was measured with the Depressive Symptom Index: Suicidality Subscale (DSI-SS; Joiner et al., 2002), a four-item measure that assessed the severity of suicidal ideation and behaviors over the previous two weeks. Each item is rated on a 4-point scale (e.g., never to always). The DSS-SI has shown excellent reliability and validity; a cut-point of 3 or higher indicates elevated levels of suicidality. Cronbach’s alpha reliability for the current sample was 0.94. Exposure to other forms of trauma was assessed with the Traumatic Events Questionnaire (TEQ; Vrana & Lauterbach, 1994), which assesses 11 traumatic events (e.g., combat, natural disasters, physical/sexual child abuse). Participants indicate whether they have ever experienced each event. The TEQ has demonstrated excellent test–retest reliability and validity, with Cronbach’s alpha for the current sample at 0.70. Finally, stigma associated with IPV experiences was assessed with the internalized (6 items) stigma subscale of the Intimate Partner Violence Stigma Scale (Crowe et al., 2019), a measure designed to assess stigma related to IPV. Participants indicate on a 6-point scale (1 = strongly disagree, 6 = strongly agree) how much they agree with each item. Initial assessments of this scale indicate that it has strong psychometric properties. Cronbach’s alpha for the current sample was 0.67.

## 3. Results

Less than 2% of the data was missing on any given item. For continuous variables, missing values were replaced with the sample mean for that specific variable. Mean substitution was used, given the small proportion of missing data and to preserve sample size for multivariable analyses. In contrast, missing values on dichotomous variables were left as missing because mean substitution for binary indicators produces non-interpretable values and can distort category membership. The prevalence of the victimization of the different forms of IPV assessed by these scales is presented at the bottom of Table 1.

### 3.1. COVID-19-Related Experiences

Of the participants, 63.5% indicated that they were subject to stay-at-home orders, with 56.6% saying they were confined to the house with their aggressive partners. On average, they were confined together for 4.99 months (*SD* = 3.46). In addition, 59.7% said the COVID-19 pandemic led to financial strain, while 55.4% said it led to additional unforeseen expenses. Of the 151 participants with children, 64.2% said that their school-aged children moved to fully remote learning, and 49.7% indicated that they had no access to childcare outside the home. Table 3 presents additional COVID-19-related experiences of the participants; 42% indicated that either they and/or their partners experienced a job loss; 57.1% indicated that either they and/or their partners experienced a change in their work schedule; 49.9% said that either they or their partners were considered essential personnel, and 30.3% indicated that their job and/or their partner’s job put them at risk for a COVID-19 infection. Finally, 17.5% said that either they and/or their partners fell ill with COVID-19 during the time period encompassing the start of the pandemic until they took the survey. Follow-up questions showed that of those experiencing a COVID-19 infection, 18.5% of the men were hospitalized, 16.7% of the partners were, and 1.9% had both been hospitalized.

### 3.2. COVID-19-Related IPV Experiences

We then asked the participants whether their IPV victimization became more frequent or severe during the COVID-19 pandemic; 16.8% said that their partner’s aggression became more severe and frequent; 18.4% said that it became just more frequent; 12.6% said that it became just more severe. Thus, 47.8% of the participants experienced an increase in either or both the frequency and severity of IPV victimization. In contrast, 37.5% indicated that their partner’s aggression stayed the same, while 14.6% indicated that their partner’s aggression got better.

Table 2 illustrates the various COVID-19-related barriers to help-seeking that the participants reported experiencing. Overall, 24.5% reported that the pandemic did not prevent them from getting assistance; thus, 75.5% reported one or more barriers, the most common of which—reported by 39.9% of participants—was not wanting to go to any in-person support groups because of concerns about getting infected with COVID-19. The second most common barrier (22.6%) was that a DV agency said they could not offer in-person support because of COVID-19 concerns, followed by not being able to escape to a family member’s or friend’s house because of concerns of contracting or spreading COVID-19 (reported by 21.4%). All barriers were endorsed by at least 35 (11%) of the participants, the most common of which were not wanting to attend in-person support groups because of concerns about COVID-19 infection (39.9%), a DV agency not offering in-person saying they weren’t offering in-person support because of COVID-19 concerns (22.6%), not being able to escape to a family member or friend’s house because of concerns about COVID-19 infection (21.4%), and not wanting to seek shelter/refuge because of COVID-19 infection concerns (20.8%). Furthermore, 4.1% indicated another barrier not listed, the most common of which was courts being difficult to access or court hearings being delayed due to COVID-19 (*n* = 4).

### 3.3. Correlates/Predictors of Increased Frequency/Severity of IPV Victimization

We then conducted a series of analyses to investigate potential correlates of an increase in frequency and/or severity of IPV victimization due to the COVID-19 pandemic. We dichotomized our measure assessing increased frequency and/or severity of IPV victimization, such that 1 indicated that the participant reported an increase in severity and/or frequency of IPV victimization (47.8%), whereas a 0 indicated the participant did not report any increase in the severity or frequency of IPV victimization (52.2%). Potential correlates of this dichotomized variable included the COVID-19-related experiences the participants reported, with the exception of hardships due to children’s care and schooling and hospitalization due to COVID-19, because of the decrease in sample size associated with including those variables. Other potential correlates included demographics, mental and behavioral health indicators, and overall IPV victimization in their relationship as measured by the variety scores. Table 4 presents the results of point-biserial correlations.

Of the COVID-19-related experiences, barriers to accessing help, job loss, work schedule changes, being subject to stay-at-home orders, being confined to the house with their aggressor, financial strain, and unexpected expenses were all significantly correlated with increased frequency/severity of IPV victimization. No demographic variables were significantly associated with increased frequency/severity of IPV victimization; however, increased frequency of alcohol use, elevated suicide risk, and number of traumatic experiences were all significantly and positively related to increased frequency/severity of IPV victimization. Finally, the variety scores of all types of IPV victimization were significantly related to increased frequency/severity.

We then entered the variables that were significant at the bivariate level into a backward stepwise logistic regression model to investigate which variables were independent predictors of increased frequency/severity of IPV victimization. A backwards logistic regression model uses a likelihood ratio test to evaluate which predicts can be removed from the model so that only unique predictors remain at an alpha level of 0.10. The final model (χ^2^ (6) = 68.80, *p* < 0.001) is presented in Table 5. Overall, this model explained 28.8% of the variance in increased frequency/severity, as indicated by the Nagelkerke R^2^. The classification statistics suggest that this model correctly classifies 70.3% of participants; 67.3% of those with no increased frequency/severity, and 73.2% of those with increased frequency/severity. This model had four significant independent predictors: help-seeking barriers, job loss, being confined to the house with their aggressor, and the number of prior traumas. Specifically, male IPV victims who reported at least one barrier to getting help were 2.56 times more likely to report that their IPV victimization increased in frequency and/or severity. Male victims who reported that they and/or their partners experienced a job loss were 1.91 times more likely to report increased frequency and/or severity. Male IPV victims who were confined to their homes with their aggressors were 3.84 times more likely to experience increased frequency/severity, while each additional traumatic experience reported resulted in an increase in odds of 1.20 for increased frequency and/or severity of IPV victimization.

### 3.4. Correlates/Predictors of Barriers to Accessing Help

We conducted the same series of analyses to investigate correlates and predictors of barriers to accessing help during COVID-19. Bivariate analyses (Table 4) showed that the following COVID-19-related experiences were significantly and positively correlated with COVID-19-related barriers to accessing help: having an increased frequency/severity of IPV, being subjected to stay-at-home orders, being confined to the house with their aggressor (marginal correlation), experiencing increased financial strain, and having unexpected expenses. Regarding demographic predictors, greater length of relationship was negatively correlated with experiencing barriers, as was parenting children together and the age of both the participant and his partner. Those who were married to their partners were significantly more likely to experience barriers. Substance use was also negatively associated with barriers to getting help: participants who reported ever using marijuana, ever misusing prescription medications, and ever using illicit drugs were significantly less likely than their counterparts to report barriers to accessing help, and participants who reported greater frequency of alcohol use also indicated they were less likely to experience barriers. Moreover, participants with greater trauma history and participants who reached a cut-off for severe depression were also less likely to experience barriers. However, the more experiences of internalized IPV stigma, the more likely they were to report barriers to getting help. Finally, the more IPV the participants reported experiencing, the more likely they were to report COVID-19-related barriers to accessing help.

A stepwise backward logistic regression model indicated that nine of the above variables were unique predictors of barriers to accessing help during COVID (χ^2^ (9) = 110.68, *p* < 0.001). The final model predicted 47.8% of the variance in experiencing barriers, according to the Nagelkerke R^2^, and the model correctly classified 85.9% of participants, including 56.3% with no barriers and 94.8% with barriers. As shown in Table 5, participants who reported that their partners’ aggression became more frequent and/or severe during COVID were 3.15 times more likely to report COVID-19-related barriers to accessing help. In addition, those who reported financial strain during COVID were 2.49 times more likely to report barriers. For each additional year in the relationship, men were 10% less likely to report barriers, while married men were 3.85 times more likely to report barriers, in comparison to all other men. Men who used marijuana and those who met the cut-off for severe depression were less likely to report barriers (83% and 86%, respectively). For each additional point they scored on the scale measuring internalized IPV stigma, they were 1.15 times more likely to report barriers. Finally, for each additional type of coercive control victimization they experienced, they were 1.17 times more likely to report barriers.

## 4. Discussion

The purpose of the current study was to explore male IPV victims’ experiences during the COVID-19 pandemic. Specifically, we explored the extent to which male IPV victims experienced increasing frequency and/or severity of IPV as a result of the COVID-19 pandemic, and the extent to which they experienced various COVID-19-related barriers to accessing help. In addition, we investigated potential predictors of increased frequency and/or severity of IPV victimization and of barriers to accessing help. Prior research (e.g., Usta et al., 2021) has focused largely on these issues among women as IPV victims during the COVID-19 pandemic. However, research also demonstrates that at least a substantial minority of IPV victims are men (Leemis et al., 2022), that most male IPV victims have female perpetrators, and that this victimization has deleterious mental health consequences (e.g., Hines & Douglas, 2015), thus there is a need to explore how male IPV victims fared during the COVID-19 pandemic to help inform prevention and intervention. Furthermore, there are additional barriers to accessing help for male IPV victims (Machado et al., 2017), including feeling internalized stigma due to their gender (Taylor et al., 2021), fearing they will not be believed by a system that still views men as primarily perpetrators (Bates, 2020), and fearing losing their children (Bates & Taylor, 2021).

### 4.1. Increases in Frequency and Severity of IPV

Almost half of the participants indicated that their partner’s aggression became either more severe and/or more frequent during the COVID-19 pandemic. This increase occurred between March 2020 and whenever the participant took the survey in 2021. Just over a third said that their partner’s aggression stayed the same, while just under 15% indicated it actually got better. This data supports what is seen in other studies that explored women’s victimization (e.g., Boxall & Morgan, 2020) and studies exploring a more inclusive range of victim groups (e.g., Thiel et al., 2022). For example, Gilchrist et al. (2023) found across a range of gender and sexual orientation groups that 38.2% of victims and 37.6% of perpetrators reported increases in IPV during the pandemic. Indeed, this increase in IPV has been mirrored in other forms of abuse such as child and elder abuse and has been linked to the stressful environment and economic instability of the pandemic (e.g., Kourti et al., 2023).

We also explored correlates of these increases in frequency/severity of aggression. Based on prior research with female IPV victimization (Ceroni et al., 2023; Gosangi et al., 2021; Lyons & Brewer, 2022; Schokkenbroek et al., 2021), we hypothesized that being subject to stay-at-home orders, being confined with one’s partner to home, barriers to getting access to support, job loss, financial strain, career strain, and mental health concerns, would be associated with increased frequency and/or severity of IPV victimization. This hypothesis was largely supported. Specifically, in the multivariate analysis, being confined with one’s aggressor to the home, barriers to accessing support, and job loss all predicted increased frequency and/or severity. In terms of mental health concerns, men with increased levels of trauma history were also more likely to report increased frequency and/or severity, although none of the measures specifically designed to assess specific forms of mental health issues were significantly associated with increased frequency and/or severity. Furthermore, although being subjected to stay-at-home orders, financial strain, and career strain (e.g., change in hours) were not unique predictors of increased frequency and/or severity in the multivariate analyses, they were associated on the bivariate level.

These analyses show that male IPV victims, like female IPV victims, were likely to experience increases in frequency and/or severity of victimization during the COVID-19 pandemic, and that these increases were due to similar reasons, such as being confined to the home and not being able to access resources for help. Indeed, this supports a range of other research that has explored the factors that increased IPV for victims during this pandemic period. These factors included being made to stay at home (e.g., Henke & Hsu, 2022), loss of jobs (e.g., Agüero et al., 2024), and other economic factors (e.g., Wood et al., 2023). Also, our findings are consistent with findings that show that although there are differences, male and female IPV victims have largely similar experiences in terms of frequency, severity, and health consequences (e.g., Hines & Douglas, 2010, 2018).

### 4.2. Barriers to Accessing Help

More than three-quarters of the participants reported one or more COVID-19-related barriers to accessing help or resources during the pandemic. These included not wanting to go to in-person support groups because of concerns about getting infected with COVID-19, a DV agency telling them that they could not offer in-person support because of COVID-19 concerns, and not being able to escape to a family member’s or friend’s house because of concerns of contracting or spreading COVID-19, among other concerns. Participants also wrote concerns about court dates being delayed due to COVID-19, a concern that may have been more widespread than assessed in this study because we did not list it as an answer choice. Nonetheless, similar to female IPV victims (e.g., Pfitzner et al., 2022), a majority of male IPV victims reported barriers to accessing services and help specifically due to COVID-19 issues.

We then conducted a series of exploratory analyses to investigate potential predictors of experiencing barriers to accessing help due to COVID-19. To our knowledge, this is one of the first studies to investigate predictors of barriers to accessing resources due to COVID-19. Significant predictors included experiencing more frequent and/or severe IPV during COVID-19 and experiencing COVID-19-related financial strain. Thus, perhaps participants who experienced more frequent and/or severe IPV were much more concerned about accessing resources during the pandemic because of the possible consequences of being discovered (e.g., not participating in telehealth support groups/treatment because their perpetrator was present), and financial strain may have made it more difficult to think about accessing IPV-related resources because of the overarching concerns about finances and perhaps fear of needing to spend additional money to access certain resources.

Men in longer relationships, however, reported being less likely to experience barriers, but conversely, married men reported being more likely to experience barriers. For married men, difficulties in accessing barriers could be due to living together, sharing children together, and sharing other resources, which may make accessing resources more difficult, particularly during a pandemic. Why there is a contradictory finding with men in longer relationships being less likely to experience barriers is puzzling and should be explored further in future research. In addition, the presence of children warrants further investigation as it is known that this is a barrier for disclosure and leaving due to fear of what will happen to the children and their relationship with them (Bates, 2020; Bates & Taylor, 2021), while other research shows that the presence of children facilitates help-seeking from certain resources, such as from a mental health practitioner, but does not predict seeking help from other resources, such as DV agencies and the police (Douglas et al., 2012).

Additional findings showed that men who used marijuana and men who were experiencing severe depression were less likely to experience barriers to resources, perhaps due to their experiences overcoming barriers to accessing help in the past for these other issues. Moreover, not surprisingly, men who reported higher scores on internalized IPV stigma were more likely to report barriers, likely due to their own internalized stigma regarding how society views IPV victims and men like them (Taylor et al., 2021). Finally, experiencing more types of coercive control victimization was related to experiencing barriers, perhaps more due to their aggressor finding various ways to control their behavior, rather than specific COVID-19-related issues.

### 4.3. Limitations

The current study has some limitations. As mentioned, all analyses were exploratory, and although some findings replicated the findings from previous studies on female IPV victims, they need to be replicated and explored further on more representative samples of male IPV victims. There are further limitations to the generalizability of our findings. There was a limited sample size in countries other than the USA, which restricted us from being able to assess potential differences across countries. Furthermore, it’s possible that some of the predictors of increased frequency/severity and experiences of barriers may be due to racial/ethnic, educational, and employment variables. Given the differences in educational and racial/ethnic compositions of the countries involved, these are potential predictors that should be further investigated within countries. Another demographic-related limitation is that we did not separately analyze heterosexual men, gay men, bisexual men, trans men, and men with other sexual and gender identities. Because of the limited sample size of gender and sexual minority men and because heterosexual sexual orientation was not significantly associated with our dependent variables, we analyzed the sample as a whole. Future research should investigate whether male IPV victims who are sexual and/or gender minorities had additional barriers to accessing resources during the peak of the COVID19 pandemic. This study also relied on men’s self-reported IPV; social desirability and other potential biases may affect the results. In addition, although the majority of men reported on victimization experiences that spanned March 2020 until they completed the survey in 2021, some may have begun their relationship after March of 2020 or ended it sometime during the peak of the pandemic, and thus future research should better capture COVID-19-related IPV and help-seeking experiences over the span of that timeline. Furthermore, different forms of IPV relationships may be associated with distinct experiences, including variation in the frequency or severity of violence as well as barriers to help-seeking. For instance, men experiencing intimate terrorism versus situational couple violence (Johnson, 2008) may face markedly different challenges relevant to the outcomes examined here. Finally, in terms of the barriers to accessing help, we did not assess whether the men wanted to access help, which could also inform whether they experienced barriers (i.e., if they did not want help, they may not have perceived barriers). Future research should explore these issues.

### 4.4. Implications

There are a number of implications that are raised through the current findings. An important implication of is that men’s experiences of IPV during the COVID-19 pandemic appear to closely mirror patterns well-documented in the literature on female victims; as discussed above, across predictors and outcomes, the associations observed in our study are largely consistent with prior COVID-19 IPV research on female victims, suggesting more convergence than divergence in experiences. Because the findings are consistent with prior research on female victims during the COVID-19 pandemic and because our study focuses on male victims, the data cannot support gender-specific interpretations. Moreover, we did not administer direct measures of gender norms, masculinity constructs, or perpetrator intent. For these reasons, we intentionally avoid speculative gender-specific interpretations.

The predominance of female perpetrators in this sample aligns with the larger literature on male IPV victims (e.g., Gilchrist et al., 2023; Leemis et al., 2022) and underscores how prevailing gendered assumptions may obscure recognition of men’s victimization without necessarily implying distinct mechanisms or impacts. Taken together, these findings highlight the need for inclusive, victim-centered IPV frameworks that recognize men’s experiences while also acknowledging substantial overlap in risk factors and outcomes across genders. Previous research highlights a number of methods to increase inclusivity in light of the pandemic, including making IPV assessment/screening tools more readily available in clinical settings (Boserup et al., 2020), supporting the development of tailored support for all victims and perpetrators (Gilchrist et al., 2023), and understanding the new barriers that the pandemic created (Pfitzner et al., 2022).

The findings from the current study point to a range of barriers to help-seeking which were positively related to the severity and frequency of IPV; it is essential that this knowledge informs the ways in which services are designed to serve all victims of IPV, including male victims. Even prior to the pandemic, evidence indicated that gender inclusive services are effective and efficient ways to deliver IPV interventions (e.g., Ensign & Jones, 2007; Hamel, 2007). It is important that post-pandemic research informs the development of interventions because the nature of service provision evolves with our new ways of working. The rapid movement of the virus and lockdowns meant that there was an urgent pivot to remote delivery of services and support (Pfitzner et al., 2022). Global prevention programs proved difficult due to a range of intersectional discriminatory factors (Kourti et al., 2023), but there is a need to understand and further explore the ways in which the pandemic affected survivors so we can make recommendations for public health and policy (Leigh et al., 2023). This would include having a strategy and protocols in place should there be any major national or international public health crises in the future.

### 4.5. Future Research Directions

Although the present study demonstrates substantial overlap between male IPV victims’ experiences during the COVID-19 pandemic and patterns previously documented among female victims, it was not designed to test gendered mechanisms or explicitly contrast gender-specific pathways into increased victimization or help-seeking barriers. As such, the findings cannot determine whether observed similarities reflect genuinely shared underlying processes across genders or whether distinct gendered processes produce convergent outcomes under crisis conditions such as a pandemic.

Future research should, therefore, explicitly engage with theoretical frameworks that address how gender may shape IPV experiences and responses to contextual stressors. For example, gender role socialization and masculinity norms may influence how men interpret, disclose, and cope with victimization, potentially affecting both perceived barriers to help-seeking and experiences of internalized stigma (e.g., Walker et al., 2020), even when objective stressors (e.g., confinement, financial strain) are similar across genders. Thus, future studies should employ designs that include multiple genders within the same analytic framework and directly test gender as a moderator of key relationships identified here, such as the associations among confinement, economic stress, and changes in IPV severity or frequency. Incorporating validated measures of gender norms, masculinity ideologies, and societal stigma would allow researchers to evaluate whether similar pandemic-related conditions are experienced, interpreted, or managed differently depending on gender.

## Figures and Tables

**Table 1:** Sample demographics (*n* = 318).

	Male Participants	Participant’s Partners
	% (*n*) or *M* (*SD*)	% (*n*) or *M* (*SD*)
**Demographics of Participants & Partners**		
Age	33.84 (7.39)	32.44 (6.90)
***Gender***		
Cismen	98.4% (313)	14.5% (46)
Ciswomen	--	83.6% (266)
Transmen	1.6% (5)	1.3% (4)
Transwomen	--	0.3% (1)
Nonbinary, genderqueer, gender nonconforming	--	0.3% (1)
***Sexual Orientation***		
Heterosexual	84.3% (268)	73.9% (235)
Bisexual/pansexual	5.7% (18)	9.7% (31)
Gay/lesbian	9.4% (30)	9.7% (31)
Other/didn’t identify	0.6% (2)	1.6% (5)
***Race/ethnicity***		
White/European/Australian	64.5% (205)	67.5% (215)
Black	14.2% (45)	8.2% (26)
Asian/East Asian/Southeast Asian	2.8% (9)	2.8% (9)
Latino/a	10.7% (34)	9.1% (29)
Aboriginal/Indigenous/Native	6.3% (21)	8.1% (26)
Pacific Islander	0.9% (3)	0.9% (3)
Middle Eastern	0.9% (3)	0.9% (3)
Other	1.3% (4)	1.3% (4)
***Education***		
Less than high school	0.9% (3)	2.2% (7)
High school	10.4% (33)	19.8% (63)
Trade school	10.7% (34)	6.3% (20)
Some college/university	17.0% (54)	14.5% (46)
College/university degree	38.4% (122)	37.5% (113)
Graduate degree	13.8% (44)	12.6% (38)
A levels (UK)	5.3% (17)	4.4% (14)
***Occupational Status***		
Full time	74.2% (236)	55.7% (177)
Part time	9.1% (29)	16.4% (52)
Unemployed & looking for work	5.0% (16)	4.7% (15)
Unemployed & not looking for work	0.6% (2)	5.0% (16)
Student	1.9% (6)	1.6% (5)
Retired	--	0.3% (1)
Homemaker	0.3% (1)	2.8% (9)
Self-employed	4.4% (14)	5.0% (16)
Unable to work	0.9% (3)	0.9% (3)
**Relationship Characteristics**		
***Relationship Type***		
Dating	9.7% (31)	
Ex-dating	8.5% (27)	
Engaged	5.7% (18)	
Ex-engaged	6.9% (22)	
Domestic partnership	8.2% (26)	
Cohabiting	11.0% (35)	
Ex-cohabiting	10.1% (32)	
Married	34.0% (108)	
Separated/legally separated	13.5% (43)	
Divorced	6.9% (22)	
Other	0.3% (1)	
Parented children together	34.0% (108)	
Relationship length (in years)	6.46 (5.46)	
***IPV Victimization During the Course of the Aggression Relationship***		
Severe Physical Abuse	86.8	
Any Physical Abuse	97.2	
Any Injury	84.0	
Severe Psychological Abuse	90.9	
Controlling Behaviors	88.4	
Legal/Administrative Abuse	74.5	

**Table 2:** COVID-19-Related Barriers to Seeking Help (*n* = 318).

Barrier	% Yes (*n*)
I didn’t want to go to any in-person support groups because I was concerned about becoming infected with COVID-19.	39.9% (127)
A domestic violence agency or preventing family harm/violence service I contacted said that they could not offer in-person support because of concerns about spreading COVID-19.	22.6% (72)
I couldn’t escape to a family member’s or friend’s house because of concerns of contracting or spreading COVID-19.	21.4% (68)
I didn’t want to seek shelter/refuge services because I was concerned about contracting or spreading COVID-19.	20.8% (66)
I didn’t want to contact the police because of concerns about COVID-19.	18.6% (59)
The only support I could find was online, but I couldn’t participate because my partner would overhear or would monitor my computer use.	15.7% (50)
I could no longer see my therapist because of concerns about contracting or spreading COVID-19.	14.5% (46)
A domestic violence agency I contacted said they couldn’t offer me shelter/refuge services because of concerns related to COVID-19.	12.2% (39)
The police didn’t want to help because of concerns about COVID-19.	11.0% (35)
The pandemic prevented me from getting assistance for some other reason.	4.1% (13)
Court access was difficult/delayed.	1.3% (4)
The pandemic did NOT prevent me from getting assistance.	24.5% (78)

**Table 3:** COVID-19 Experiences (*n* = 318).

	% Yes, I Did/Was	% Yes, My Partner Did/Was	% Yes, We Both Did/Was	% No, Neither of Us Did/Was
Experienced a job loss	17.5	15.9	8.6	58.1
Experienced a change in work schedule	20.4	14.7	22.0	42.8
Considered essential personnel	22.7	10.4	16.8	50.2
Job put at risk for infection	13.6	6.3	10.4	69.6
Fell ill with COVID	7.3	7.0	3.2	82.6

**Table 4:** Correlates of Increased Frequency and Severity of IPV during COVID-19 and with Experiences of COVID-19-Related Barriers to Seeking Help (*n* = 318).

	Correlation with Increased Frequency and Severity of IPV Due to COVID-19		Correlation with Experiences of COVID-19-Related Barriers to Seeking Help	
	*r*	*p*	*r*	*p*
***COVID-19-Related Variables***				
Experienced COVID-19 Barriers to Seeking Help	**0.21**	**<0.001**	--	--
Increased frequency & severity of IPV	--	--	0.21	<0.001
Any job loss for him or partner	**0.21**	**<0.001**	0.08	0.152
Any work schedule changes for him or partner	**0.14**	**0.012**	−0.05	0.415
Either of them essential personnel	0.08	0.155	−0.07	0.260
Either of their jobs put them at risk for infection	0.08	0.176	−0.10	0.099
One or both fell ill with COVID-19	0.05	0.349	0.05	0.388
Subject to stay-at-home order	**0.30**	**<0.001**	**0.18**	**0.001**
Confined to the house with their aggressor	**0.32**	**<0.001**	0.11	0.055
Length of time confined to home	−0.01	0.918	−0.14	0.072
Put financial strain on them	**0.23**	**<0.001**	**0.29**	**<0.001**
Led to unexpected expenses	**0.17**	**0.002**	**0.23**	**<0.001**
***Demographics***				
Relationship length	−0.06	0.327	**−0.28**	**<0.001**
Parented children together	−0.05	0.360	**−0.12**	**0.035**
Married	0.04	0.509	**0.13**	**0.020**
Age	−0.02	0.467	**−0.26**	**<0.001**
Heterosexual sexual orientation	−0.05	0.357	0.05	0.357
Partner’s age	−0.01	0.867	**−0.25**	**<0.001**
Partner cis-female	−0.04	0.469	0.04	0.469
***Mental and Behavioral Health***				
Used marijuana	0.09	0.120	**−0.26**	**<0.001**
Misused prescription medications	−0.01	0.804	**−0.12**	**0.040**
Used illicit drugs	0.04	0.797	**−0.21**	**<0.001**
Frequency of alcohol use	0.10	0.081	**−0.15**	**0.010**
Frequency of binge drinking	0.07	0.235	0.03	0.655
Frequency of intoxication	0.08	0.175	0.06	0.286
PHQ Depression Score	0.08	0.166	0.04	0.531
PHQ Severe Depression	0.07	0.256	**−0.13**	**0.030**
DSISS Elevated Suicide Risk	0.11	0.051	−0.08	0.170
TEQ Score Trauma history	**0.21**	**<0.001**	−0.11	0.051
IPVSS Experiences of Internalized Stigma	0.08	0.175	**0.31**	**<0.001**
***Intimate Partner Violence Variety Scores—Victimization***				
Any physical abuse	**0.26**	**<0.001**	**0.19**	**0.001**
Injuries	**0.25**	**<0.001**	**0.25**	**<0.001**
Severe psychological abuse	**0.25**	**<0.001**	**0.13**	**0.024**
Coercive control	**0.20**	**<0.001**	**0.18**	**0.002**
Legal/administrative abuse	**0.19**	**<0.001**	−0.04	0.474

*Note.* Bolded correlation coefficients are significant.

**Table 5:** Backward Logistic Regression Results Predicting COVID-19-Related Increased Frequency/Severity of IPV and Barriers to Seeking Help.

Independent Variable	*B*	*SE*	Wald	*p*	*OR*
***Predicting Increased Frequency/Severity of IPV during COVID (n = 283)***					
COVID-19 help-seeking barriers	0.94	0.33	8.22	0.004	2.56
COVID-19 job loss	0.65	0.28	5.26	0.022	1.91
COVID-19 confined to house with aggressor	1.35	0.29	21.62	<0.001	3.84
TEQ Score	0.18	0.07	7.19	0.007	1.20
Variety Score—Severe Psychological Abuse Victimization	0.21	0.12	3.09	0.079	1.24
Variety Score—Legal/Admin Abuse Victimization	0.14	0.08	2.90	0.089	1.15
***Predicting Barriers to Seeking Help during COVID (n = 284)***					
Aggression more frequent/severe during COVID	1.15	0.39	8.82	0.003	3.15
COVID led to financial strain	0.92	0.36	6.28	0.012	2.49
Relationship length (in years)	−0.10	0.05	5.06	0.025	0.90
Married	1.35	0.47	8.20	0.004	3.85
Age	−0.05	0.03	2.83	0.093	0.95
Uses Marijuana	−1.79	0.43	17.34	<0.001	0.17
Severe Depression	−1.94	0.62	9.72	0.002	0.14
Internalized Stigma	0.14	0.04	14.40	<0.001	1.15
Variety Score—Control Victimization	0.16	0.06	6.73	0.010	1.17

Note. Model fit statistics for predicting increased frequency/severity of IPV: χ^2^ (6) = 68.80, *p* < 0.001, Nagelkerke R^2^ = 0.288. Model fit statistics for predicting barriers to seeking help: χ^2^ (9) = 110.68, *p* < 0.001, Nagelkerke R^2^ = 0.478.

## Data Availability

The dataset from the study will be made available on reasonable request by contacting the first author after all manuscripts have been published.

## Abbreviations

The following abbreviations are used in this manuscript:

IPV	Intimate partner violence
NISVS	National Intimate Partner and Sexual Violence Survey
WHO	World Health Organization
CTS2	Revised Conflict Tactics Scales
PMWI	Psychological Maltreatment of Women Inventory
PHQ9	Patient Health Questionnaire
DSS-SI	Depressive Symptom Index Suicidality Subscale
TEQ	Traumatic Events Questionnaire
